# Different Effect of Vitamin E or Hydroxytyrosol Supplementation to Sow’s Diet on Oxidative Status and Performances of Weaned Piglets

**DOI:** 10.3390/antiox12081504

**Published:** 2023-07-27

**Authors:** Gerardo Gómez, Hernan D. Laviano, Juan M. García-Casco, Rosa Escudero, María Muñoz, Ana Heras-Molina, Antonio González-Bulnes, Cristina Óvilo, Clemente López-Bote, Ana I. Rey

**Affiliations:** 1 Instituto Regional de Investigación y Desarrollo Agroalimentario y Forestal de Castilla-La Mancha (IRIAF), Ctra. Toledo-Albacete s/n., 13700 Tomelloso, Spain; 2 Departamento Producción Animal, Facultad de Veterinaria, Universidad Complutense de Madrid, Avda. Puerta de Hierro s/n., 28040 Madrid, Spain; 3 Departamento de Mejora Genética Animal, Instituto Nacional de Investigación y Tecnología Agraria y Alimentaria, INIA, CSIC, Ctra Coruña km 7.5, 28040 Madrid, Spain; 4 Departamento de Producción y Sanidad Animal, Facultad de Veterinaria, Universidad Cardenal Herrera-CEU, CEU Universities, C/Tirant lo Blanc, 7, Alfara del Patriarca, 46115 Valencia, Spain

**Keywords:** piglet’s growth, Iberian sows, body measurements, antioxidants, olive derivatives

## Abstract

Different feeding strategies are being applied to sows in order to obtain homogeneous piglets’ weights and improved health status. This study evaluated how the dietary supplementation of vitamin E (VE) (100 mg/kg), hydroxytyrosol (HXT) (1.5 mg/kg) or the combined administration (VE + HXT) given to Iberian sows from day 85 of gestation affected the growth pattern of the piglets and their oxidative status; and quantified what these effects were due to. Dietary VE and HXT improved the oxidative status of sows and piglets. Both VE and HXT modified the growth pattern at birth and performances of the piglets in a different way according to the growing period. Piglets’ performances were positively correlated with plasma VE and negatively with plasma malondialdehyde (MDA) of the sow. However, the highest variation in growth patterns was explained by the colostrum composition. Significant linear equations were observed between piglets’ performances and colostrum saturated (SAT), n-7 monounsaturated fatty acids (C16:1n-7 and C18:1n-7) and different desaturases indices. This study would confirm that VE supplementation to the sow diet could be more adequate than HXT for the improved development during the first weeks of a piglet’s life. The combined administration of both antioxidants would not produce additional positive effects compared to the individual supplementation.

## 1. Introduction

The increase in sows’ prolificacy leads to a greater number of low-weight piglets and heterogeneity in the litter [1,2]. This can be especially problematic in the case of traditional and fatter breeds, such as the Iberian pig, because of their lower prolificacy [3] and their smaller uterus, which could affect the piglet’s size to a greater extent [4]. A low piglet body weight can compromise survival and alter developmental patterns, health status and later the quality of commercial traits [2,5]. In addition, the high nutrient demand by the mother during the last period of gestation and lactation, which implies an increase in the catabolic and oxidative state, that can lead to the appearance of metabolic and immune problems in the offspring [6]. Moreover, weaning is especially problematic for piglet homeostasis, causing an increase in oxidative stress and mitochondrial dysfunction that is more severe in low-weight piglets [7]. Therefore, the use of antioxidant supplements given to the mother during these critical periods has been proposed in order to reduce the imbalance between antioxidant and oxidant substances with positive effects on the litter [8,9,10,11].

Specific studies carried out to investigate the possible effects of vitamin E (VE) supplementation in sows’ diet from day 107 of gestation until weaning reported an improvement on the antioxidant activity of sows and piglets, greater piglet weight at weaning and average daily gain (ADWG) [12]. Also, Mavromatis et al. [13] observed greater piglet weights at weaning after dietary administration of 70 mg/kg VE plus 0.45 mg Se/kg during gestation. Additionally, Mahan et al. [14] and Amazan et al. [9,10] reported that sows’ supplementation with VE during lactation is an effective strategy to improve the health status, oxidative stress of piglets and VE concentrations after weaning.

There is also previous evidence on the possible effects of olive-derived antioxidant administration to sows on piglets’ performances. Hence, positive effects on newborns’ viability and piglets’ growth have been reported after hydroxytyrosol supplementation to sows from day 35 of pregnancy [15]. However, there is no information on the consequences of its administration during other phases of pregnancy.

Despite the positive effects described for both antioxidants on the growth and development of the piglet, there is a lack of information comparing their effectiveness or that of their combined administration (one with lipophilic and the other with hydrophilic characteristics), or trying to explain what their effects are due to. The only existing investigations to our knowledge have been carried out in piglets [16] or fattening pigs [17] by the administration of another hydrophilic olive derivative (oleuropein) or VE without studying the effect of administration to the mother. Moreover, these studies were mainly focused on the comparison of the oxidative stress parameters without delving into the effects on piglet development.

Therefore, it is hypothesized that the combined administration of both antioxidants from the gestation period could maintain a more adequate state of homeostasis, reducing the negative effects associated with oxidative stress during the last reproductive phase of the sow and weaning of the piglet, with positive consequences for piglet growth.

The aim of the present study was firstly, to evaluate how the dietary supplementation of vitamin E (VE) (100 mg/kg), hydroxytyrosol (HXT) (1.5 mg/kg) or both (VE + HXT), supplemented to Iberian sows’ diet from gestation day 85, affect piglets’ growth pattern and oxidative status; and secondly, quantify the maternal effect (sow’s oxidative status or milk composition) on the piglets development until weaning.

## 2. Materials and Methods

### 2.1. Chemicals

All chemicals used were analytical grade and were supplied by Sigma-Aldrich (Alcobendas, Madrid, Spain), Panreac (Castellar del Vallès, Barcelona, Spain) or Scharlau (Sentmenat, Barcelona, España).

### 2.2. Ethics Statement

Experimental procedures related to animal handing and welfare were in compliance with the Spanish Policy for Animal Protection (RD53/2013) [18], and European Union Directive 2010/63/UE [19] for the care and use of animals in research. The INIA Committee of Ethics in Animal Research approved the experimental procedures (report ORCEEA 2019-10). The experiment was carried out at the animal facilities of Dehesón del Encinar (Oropesa, Toledo, Spain) that are in accordance with the requirements for Scientific Procedures Establishments.

### 2.3. Animals, Experimental Procedures and Diets

This research involves a total of fifty Iberian sows (half primiparous and half multiparous with between 4–5 parity) (107.2 ± 29.8 kg) that were pregnant by natural mating (Dehesón del Encinar, Oropesa, Toledo). At the time of mating, all pigs were adequately immunized according to the farm’s vaccination program. The research was conducted from August 2020 to March 2021. During the pre-experimental period (until day 85 of pregnancy), sows were given a standard grain-based diet (g/kg: 888 dry matter, 124.6 protein, 29.9 fat, 49.3 fiber, 62.1 ash; and 3050 kcal/kg Metabolizable Energy) (Sanchez Romero Carvajal, Spain). At day 85 of pregnancy (126.2 ± 29.3 kg), sows were weighted and divided into four homogeneous experimental groups (n = 12–13 per dietary treatment with equal distribution of primiparous and multiparous) and started receiving four different experimental diets until weaning (28 days). The experimental diets were supplemented with different levels of antioxidants (vitamin E or hydroxytyrosol) as follows: (1) control group: 30 mg of α-tocopheryl acetate/kg feed; (2) VE group: 100 mg of α-tocopheryl acetate/kg; (3) HXT group: 30 mg α-tocopheryl acetate/kg and 1.5 mg hydroxytyrosol/kg and (4) VE + HXT: 100 mg/kg of α-tocopheryl acetate/kg and 1.5 mg hydroxytyrosol/kg feed. During this experimental period (from day 85 of gestation until day 28 of lactation) feed administration was adjusted to fulfill daily maintenance requirements according to the National Research Council (NRC) [20] (Table A1, Appendix A). In order to comply with the α-tocopherol levels recommended by the NRC [20] for breeding sows and taking into account a contribution of about 12–14 mg/kg by the dietary ingredients, a dose of 30 mg α-tocopherol/kg feed was used for basal diet supplementation. The HXT supplementation dose was based on previous studies [15]. The α-tocopheryl acetate used in the diets was obtained from DSM Nutritional Products (Alcalá de Henares, Madrid, Spain) and the hydroxytyrosol extract (Olea europaea L. dry extract, N20130102 containing a minimum of 1.5% of hydroxytysol confirmed by analysis of the supplier) was obtained from Natac (Alcorcón, Madrid, Spain).

One week before the expected farrowing day, sows were moved from the gestation unit to the farrowing pens (one sow per pen). Each farrowing pen was provided with a farrowing crate to avoid crushing, an infrared lamp and adequate dry bedding in a localized area for piglets to reach the adequate comfort environment (at least 30 °C). At birth, live piglets (averaged litter size of 7–8) were tagged with ear tags, intramuscular iron was injected behind the ear (Previron 200, Hipra, Talavera de la Reina, Toledo, Spain) to avoid anemia problems and the tail was cut. All facilities and management were in compliance with RD 53/2013 [18]. Piglets remained with the sow until weaning.

### 2.4. Growth Evaluation

Sows were weighted again after farrowing and at weaning, whereas piglets’ (n = 344) weights were recorded at birth, at days 7, 20, at weaning (28 days) and afterwards, every month approximately during a period of 3 months. Thus, the corresponding average daily weight gain (ADWG) for each piglet was calculated during different periods as the difference between the final weights minus the initial weight divided by the number of days elapsed.

At birth (first 24 h of life), morphological measurements of piglets (biparietal diameter, occipito-nasal length, trunk length, thoracic perimeter, and abdominal perimeter) were taken by means of a caliper and a measuring tape. Additionally, one male piglet per litter was selected and euthanized 5 days post-weaning in compliance with RD 53/2013 [18] (n = 12 per dietary treatment). Then, the same measurements described above and different weights of the body and organs (total body weight, carcass, bowels, head, loin, liver, kidneys and gut) were taken.

### 2.5. Sample Collection

Blood samples (5 mL) were collected in sterile EDTA vacuum tubes (Vacutainer, BD, Franklin Lakes, NJ, USA) from a representative number of sows (n = 7 per experimental treatment) at day 110 of gestation and 20 days of lactation, whereas piglets blood samples (n = 7–8 per treatment) were extracted at day 20 of lactation and 5 days post-weaning. To obtain plasma, blood samples were immediately centrifuged at 2500 rpm for 10 min, transported in dry ice and kept at −80 °C until analysis (less than 1 month).

### 2.6. Laboratory Analysis

#### 2.6.1. Antioxidant Enzymes Determination

Analysis of antioxidant enzymes: superoxide dismutase (SOD), and different forms of glutathione: total glutathione (GSHt), oxidized (GSSG) and reduced (free GSH) was carried out spectrophotometrically (Multiscan ScanGo, Thermo-Fisher Scientific, Alcobendas, Spain) with commercial kits (Arbor Assays, Ann Arbor, MI, USA) according to the manufacturer’s instructions. The concentrations of the different forms of glutathione were measured using the same glutathione colorimetric kit. Free glutathione (free GSH) concentrations were obtained by subtracting the GSSG levels (obtained from the 2-vinylpyridine-treated standard and samples), from non-treated standards and samples (GSHt). The concentrations were calculated as µM of glutathione. SOD was expressed as U/mL.

#### 2.6.2. Tocopherol Quantification in Plasma Samples

The Vitamin E (α-tocopherol) concentration in sows’ and piglets’ plasma was extracted by the direct procedure described by Rey et al. [21]. Briefly, dibasic sodium phosphate buffer (0.054 M) adjusted to pH 7.0 was added to duplicate plasma aliquots. Tocopherol was extracted by centrifugation (600× *g* during 10 min at 4 °C) after the addition of absolute ethanol and hexane. Then, the upper layer was collected and evaporated by N_2_ stream and the remaining residue was dissolved in ethanol and injected into an HPLC (HP 1200, equipped with a diode array detector and a reverse RP-18 column) (Agilent Technologies, Waldbronn, Germany) [21]. Identification and quantification were carried out using the pure compound (Sigma-Aldrich, Alcobendas, Madrid). Results were expressed as µg of α-tocopherol per mL of plasma.

#### 2.6.3. TBARs Quantification in Plasma Samples

The thiobarbituric acid reactive substances (TBARs) in plasma samples of sows and piglets were measured as the reaction products of malondialdehyde (MDA) with thiobarbituric acid as described elsewhere [9,10]. Absorbance was read spectrophotometrically at 532 nm (Multiscan ScanGo, Thermo-Fisher Scientific, Alcobendas, Spain). MDA concentrations were calculated using 1.56 × 10^5^ M^−1^ × cm^−1^ as the molar absorption coefficient. Results were expressed as mmoles MDA/L plasma.

### 2.7. Statistical Analysis

Data of piglets’ performances (0–58 days) and body measurements at birth were analyzed following the proc MIXED of SAS (version 9.4; SAS Inst. Inc., Cary, NC, USA) and the sow was considered the experimental unit. However, for analysis of sow parameters or piglets post-weaning (1 piglet selected per litter), the general linear model (GLM) procedure contained in SAS (version 9.4; SAS Inst. Inc., Cary, NC, USA) was used. In this case, the individual sow or piglet was considered the experimental unit. Comparison between means was conducted using the Duncan test. Data are presented as the mean of each group and root mean square error (RMSE) together with the significance levels (*p* value). Pearson correlation coefficients and regression equations between oxidative parameters of sows and piglets’ measurements post-weaning were carried out using the Statgraphics-19 program. Regression equations between growth of post-weaning piglets and colostrum or milk composition (analysed by Laviano et al.) [22] were also built by the Statgraphics-19 program. Differences were considered statistically significant when *p* < 0.05, whereas *p* > 0.05 and <0.1 were considered as trends.

## 3. Results

### 3.1. Oxidative Status of Sows and Piglets

The oxidative status of sows given VE or HXT from day 85 of gestation is presented in Table 1. Regarding the gestation period, sows given a VE-supplemented diet at 100 mg/kg had higher plasma VE levels (*p* = 0.004) than groups with 30 mg/kg of VE; however, no significant changes on antioxidant enzymes at day 110 of gestation were observed. HXT supplementation improved the oxidative status of sows during gestation by the increase in SOD (*p* = 0.044) activity, GSHt (*p* = 0.019) and free GSH (*p* = 0.028) concentrations. During lactation, changes in the antioxidant’s enzymes by the dietary supplementation were not observed; however, plasma vitamin E concentration was greater (*p* = 0.0001) in VE-supplemented groups at day 20 of lactation.

**Figure 1:**
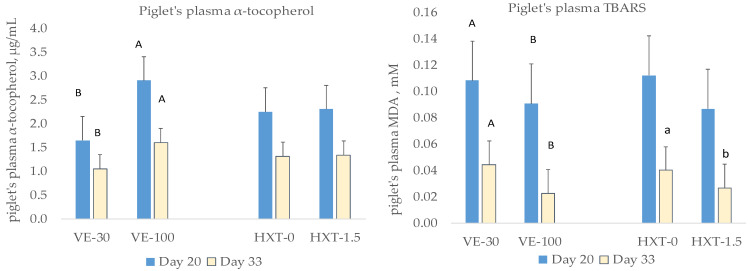
Oxidative status of piglets pre- or post-weaning from sows given α-tocopheryl acetate (VE: 30 vs. 100 mg/kg) or hydroxytyrosol (HXT: 0 vs. 1.5 mg/kg) from day 85 of gestation. ^a,b,A,B^ Letters with different superscripts were statistically significant.

The oxidative status of piglets was also evaluated at day 20 of lactation and 5 days post-weaning (33 days) (Figure 1). Piglets from VE-supplemented sows had lower MDA concentration at day 20 (*p* = 0.030) and at day 33 (*p* = 0.002). HXT supplementation to sows also reduced the MDA production in piglets at day 33 post-weaning (*p* = 0.046). The combination of both antioxidants did not produce any synergistic effect on the piglet’s parameters of oxidative stress when compared to the independent administration of the antioxidants to the mother.

### 3.2. Performances and Piglet’s Growth

The performance parameters of sows and piglets as affected by VE or HXT supplementation are shown in Table 2. Sows’ performances (body weight through gestation and lactation) were not significantly affected by the antioxidant’s supplementation. However, piglets born from sows given VE or HXT in their diets had a higher birth weight (BW) (*p* < 0.05) compared to those born from non-supplemented sows. Moreover, dietary supplementation with VE or HXT produced a significant increase in piglets’ weight at day 7 of age (*p* < 0.05). However, from 8 to 20 days of age, differences in the piglets’ weight by the antioxidant’s supplementation was not as marked, and the VE + HXT group tended to have the lowest body weight (interaction effect, *p* = 0.062) at day 20 of age.

The weight differences up to day 28 resulted in differences in the ADWG according to dietary treatments. Hence, HXT supplementation to sows produced higher ADWG during the first week of a piglet’s life (*p* = 0.042) than the non-supplemented groups. However, from 8 to 20 days, the ADWG reached the lowest values in those piglets born from HXT-supplemented sows (*p* = 0.001), and the combined administration with VE did not increase ADWG when compared with the groups given the antioxidants individually (interaction effect, *p* = 0.017). During the period from 21 to 28 days (before weaning), piglets born from VE sows had higher ADWG (*p* = 0.0001) when compared to those from non-supplemented sows. Afterwards (from 29–58, from 59–84 and from 85–110 days), ADWG was not affected by the antioxidant supplementation to the sow diet.

Piglets’ body measurements were also affected by the dietary supplementation of sows with VE or HXT (Table 3). The VE and HXT supplements independently increased the biparietal diameter (BPD) (both *p* < 0.001), trunk length (TL) (*p* = 0.023 and *p* < 0.001, respectively), body to head ratio (*p* = 0.042 and *p* = 0.007), thoracic perimeter (TP) (*p* = 0.004 and *p* < 0.001), and abdominal perimeter (AP) (*p* < 0.001 and *p* < 0.006). The VE supplement increased the occipital-nasal length (ONL) (*p* < 0.001), while HXT had no effect (*p* = 0.609). There was an interaction between VE and HXT that the affected body to head ratio (*p* = 0.003), with both supplements having a higher ratio than controls (all, *p* < 0.05). A similar interaction affected trunk length (TL) (*p* = 0.014), with it longer for the HXT and VE + HXT treatments compared to the VE treatment and all longer than length for control piglets (all, *p* < 0.05).

After weaning, differences in the growth response of piglets to sows’ antioxidant supplementation was not as marked as at birth (Table 3). A tendency to have a higher carcass weight (*p* = 0.073) and body to head ratio (*p* = 0.083) was only observed in those piglets supplemented with VE. Also, piglets from HXT-supplemented sows showed a tendency to have higher BPD (*p* = 0.084). The other measurements of the body or weight of the organs of the piglets after weaning were not significantly affected by the dietary treatment of the mother.

### 3.3. Relationship between Sows’ Oxidative Status and Piglets’ Growth

**Figure 2:**
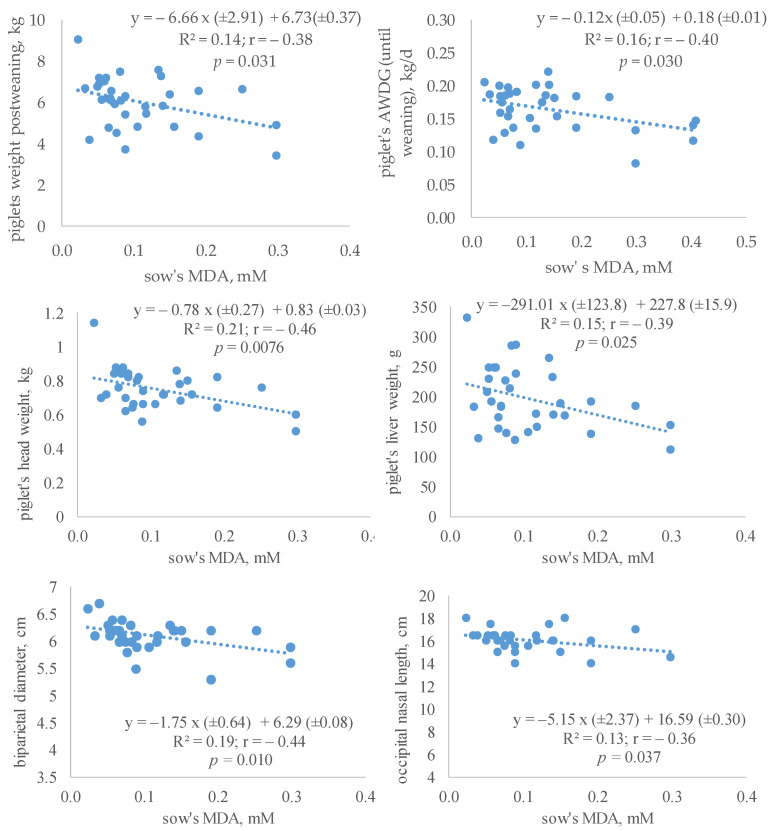
Regression equations between oxidative status of sows (MDA concentration) and growth of post-weaning piglets born from sows given α-tocopheryl acetate (VE: 30 vs. 100 mg/kg) or hydroxytyrosol (HXT: 0 vs. 1.5 mg/kg) from day 85 of gestation. ADWG = average daily weight gain.

The relationship between the oxidative parameters of sows at day 20 of lactation and piglets’ measurements post-weaning are presented in Table 4 and Figure 2. Piglets’ ADWG was positively correlated with sows’ plasma vitamin E concentration (r = 0.36, *p* < 0.05) and negatively with MDA content (r = −0.40, *p* < 0.05). Piglets’ body weight post-weaning, TP and AP were also negatively correlated with sows’ GSSG (r = −0.41, *p* < 0.05), whereas loin and kidney weights were negatively correlated with sows’ MDA concentration (r = −0.40, *p* < 0.05). Liver and head weights negatively correlated with both parameters, GSSG (r = −0.39, *p* < 0.05) and MDA (r = −0.38, *p* < 0.05 and −0.47, *p* < 0.001, respectively). BPD and ONL also correlated negatively with GSSG (r = −0.48, *p* < 0.001 and r = −0.46, *p* < 0.05, respectively) and with MDA (r = −0.45, *p* < 0.05 and r = −0.38, *p* < 0.05, respectively). The highest correlations were those related to BPD measurements and the weight of the head (r = −0.48 and −0.47, *p* < 0.001, respectively).

Carcass weight, bowels weight, gut weight and trunk length did not correlate with any of the antioxidant parameters.

The relationships between sows’ plasma GSSG and MDA and piglets’ traits followed a significant linear adjustment. Some of these relationships were quantified by regression equations and are presented in Figure 2. The linear fix varied from R^2^ = 0.13 (for ONL) to R^2^ = 0.21 (head weight).

### 3.4. Relationship between Milk Composition and Piglet’s Growth

Pearson correlation coefficients and regression equations between colostrum and milk composition (evaluated in a previous paper by Laviano et al. [22]) from supplemented sows’ and piglets’ measurements post-weaning are presented in Table 5. Significant linear relationships were observed between ADWG and colostrum saturated fatty acids (SAT) (r = −0.69; *p* < 0.001), C18:0 (r = −0.62, *p* = < 0.001), C16:1 n-7 (r = 0.57, *p* = 0.002), C18:1 n-7 (r = 0.48, *p* = 0.012), and different desaturase indices (*p* < 0.05). Loin, liver and gut weights were also linearly related with different elongase (*p* < 0.05) and desaturase (*p* < 0.05) indices of colostrum; as well as the TP and AP (*p* < 0.05). The highest correlations and linear adjustments were observed between ADWG (0–28 days) and C18:1 n-9/C18:0 ratio (r = 0.70, R^2^ = 0.49), followed by SAT (r = −0.69, R^2^ = 0.47) and C16:1 n-7/C16:0 (r = 0.63, R^2^ = 0.40). Also, high correlations and linear regressions were observed between ONL post-weaning and polyunsaturated fatty acids (PUFA), mainly n-3 (r = 0.51, R^2^ = 0.26, *p* = 0.006); TL was negatively and significantly related with colostrum PUFA, especially n-6 fatty acids (r = 0.51, R^2^ = 0.16).

Milk composition at day 20 of lactation was also related with piglets’ growth patterns at weaning (Table 6). Thus, a positive and linear relationship was observed between elongase C20-C18 and piglets’ weight post-weaning (r = 0.45, R^2^ = 0.21, *p* = 0.020), head weight (r = 0.49, R^2^ = 0.24) and trunk length (r = 0.41, R^2^ = 0.17, *p* = 0.036). However, AP and gut weight were mainly affected by the C18:3 n-3 proportions.

**Figure 3:**
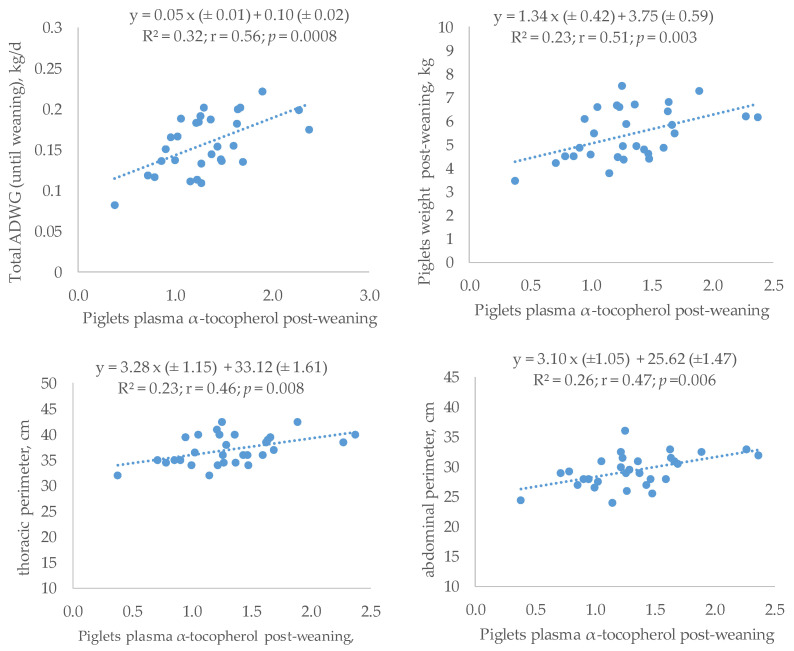
Regression equations between plasma α-tocopherol (µg/mL) and growth parameters (weight and averaged daily weight gain (0–28 d): ADWG) or measurements of weaned piglets (day 33) born from sows given α-tocopheryl acetate (VE: 30 vs. 100 mg/kg) or hydroxytyrosol (HXT: 0 vs. 1.5 mg/kg) from day 85 of gestation.

Finally, positive linear adjustments were observed between piglets’ plasma VE level and performances (Figure 3): ADWG (r = 0.56; R^2^ = 0.32; *p* = 0.0008) and weight (r = 0.51; R^2^ = 0.23; *p* = 0.003). Also, plasma VE concentration was directly and significantly related with the growth of piglets. Specifically, significant regression equations were found between the plasma VE and TP (r = 0.46; R^2^ = 0.23; *p* = 0.008) and AP (r = 0.47; R^2^ = 0.26; *p* = 0.006).

## 4. Discussion

Achieving an adequate weight at birth and a good development of the piglet are relevant aspects in modern pig production since this will determine that the animal is in a better state to face periods of high stress such as weaning. Considering the previous observed positive effects of antioxidants on piglets’ growth, and since it was intended to evaluate the possible additive effect of these compounds (VE or HXT), the present study used similar or lower doses than those described in the research literature as effective. To our knowledge, this is the first trial in which the effects of the separated or combined maternal administration of VE or HXT on piglets’ performances have been investigated.

### 4.1. Oxidative Status of Sows and Piglets

Gestation and lactation periods are characterized for having decreased availability of antioxidants and an excessive free radical’s production [6,23,24] that could have a negative effect on sow performance. In the present study, dietary VE or HXT supplementation improved the oxidative status of sows and piglets to a different extent. The plasma VE concentration was higher in VE-supplemented sows than in controls, in both periods (gestation and lactation) and these values were within the range observed in the literature [8,9,10]. Some authors have found that plasma VE concentration suffers a decrease during late gestation (from days 90–110) [6] that was attributed to the high nutrient demand of the fetus and to a preferential transfer to them, although in a limited way, through the placenta. In the present study, as a result of VE supplementation to sows, a higher concentration of VE was observed in the supplemented piglets both at 20 days of lactation and after weaning that resulted in a reduction of their oxidative stress. The maternal supplementation with VE has been reported by other authors as an effective strategy to improve the VE levels and oxidative status of piglets mainly after weaning due to the transfer of VE through milk [8,9,10,12,14].

In a different way, the administration of HXT to sows increased the level of antioxidant enzymes during gestation. However, this effect of HXT during gestation was not observed during lactation. In any case, piglets from sows supplemented with HXT showed a reduction in MDA production post-weaning, which would indicate a maternal antioxidant effect. Other investigations reported the positive effects of polyphenols to reduce the placental oxidative status [25] and then limiting placental injury, which could alleviate the piglet’s oxidative stress [26,27,28]. The olive-derivatives’ antioxidant effect has been explained by contributing to the glutathione route and other antioxidant enzymes [17,29].

Despite the positive effects of the independent supplementation of VE or HXT, a combined administration did not produce any additive effect on the oxidative stability of the sows and the offspring. Other investigations, on the contrary, have shown a greater antioxidant effect when VE was combined with polyphenols [17,27], although these authors did not use HXT, and there are no studies comparing the combined administration of both compounds during pregnancy.

### 4.2. Performances and Piglet’s Growth

Regarding the effects on growth, dietary supplementation with antioxidants (VE or HXT) increased piglets’ weight at birth and at day 7 of age. Even though placental transfer of VE is low [30], several studies provide evidence as to the benefits on the piglets’ performances. Thus, Wang et al. [12] found that supplementation of the maternal diet with 250 IU/kg VE from day 107 of gestation improved the ADWG and weaning weight of piglets. Moreover, other studies [13] using lower doses of VE than those used in the present study (50 mg/kg), at 30, 60 and 90 days of gestation, observed an increase in piglet weight at weaning, although no effect was observed on piglets’ weight at birth. Even the parenteral administration of VE (150 mg/kg + 0.3 mg/kg) administered 7 and 21 days before farrowing has been observed to increase the piglets body weight at birth and ADWG between 1–21 days [31]. However, these authors did not clearly identify the possible reason for the effects on piglets’ growth, but they attributed these effects to the protection of the immune status [13], or to an improvement in the oxidative status of the animal [12], although these relationships were not directly proven.

Considering the hypothesis that the antioxidant status of the mother and piglet could be responsible for this improvement in litter growth, it would be expected that different antioxidants could have a similar effect. However, it is interesting to remark that the administration of polyphenols in the present study as HXT from the late gestation period, although producing an increase in piglets’ weight at birth, there was a decrease in weight of the animals during the first growing period. This effect was therefore contrary to that observed in piglets born to mothers supplemented with VE that gained weight until weaning and presented a greater ADWG during lactation. In a study in which a herbal-based antioxidant supplement containing polyphenols was used, there was no change in weaning weight, although there was a 7.4% increase in weaning weight in piglets with birth weights greater than 1 kg [32]. There is hardly any information on the use of HXT on piglet size, but some previous research reveals that HXT administration from early gestation can counteract intrauterine growth restriction and low body weight due to its improvement in placental function [15]. However, these effects were mainly observed in the larger litters (9–10 piglets); whereas ADWG decreased in piglets aged 15–25 days in litters with 2–8 neonates, similarly to what was observed in the present study. This weight loss could be explained by the different modification that VE or HXT produces in the composition of milk and colostrum [22]. Hence, sows supplemented with HXT mobilized a high amount of unsaturated fatty acids to colostrum, reducing the mother’s ability to desaturate [22]. Olive derivatives have been described as producing a higher utilization of PUFA [17], and a higher metabolic use and mobilization of C18:2 during lactation has been related to lower fat and energy in milk [22,33], which could have favored a lower weight gain compared to the non-supplemented groups.

In addition, the maternal antioxidants supplementation affected fetal development. Thus, both supplements produced piglets at birth with greater measures related to the head and trunk, and consequently larger size. Previous investigations reported the effectiveness of maternal HXT supplementation given from day 35 of gestation to improve the developmental pattern of piglets [15,34]. Results of the present research indicate that the administration of HXT from late gestation, as well as VE supplementation would also be an effective strategy. Furthermore, antioxidants supplementation seems to improve the body weight to head size ratio, which has been positively associated with post-weaning performances [35]. This effect was observed at birth for VE and HXT groups and a tendency was observed for VE group at weaning. Unexpectedly, the combined administration of the two antioxidants did not produce any additive effect, and both the development pattern of the animals and the weight gains seemed to be similar to those observed when the antioxidants were provided independently.

### 4.3. Relationship between Sows’ Oxidative Status or Milk Composition and Piglets’ Growth

It is interesting to highlight that the contribution of the oxidative status of the mother, as well as the composition of the sow’s milk during lactation on the piglet’s growth and development, has also been quantified in the present study. The fact that the oxidative status of the sow contributed significantly to the piglet’s growth is in agreement with the study of Zhao and Kim [36]. These authors observed a negative correlation of some sows’ oxidative stress indicators (MDA concentration) on day 3 of lactation on the piglet’s weight at day 18 of lactation. To our knowledge, there is no further information on sows. In a different study not carried out on breeding females but in broilers, De-Cara et al. [37] found linear relationships between plasma MDA or some antioxidant enzymes and the weight gain during the growing period. An imbalance between antioxidants/prooxidants could alter metabolic homeostasis and nutrient utilization [38], which would affect performances.

However, in the present study the greatest contribution to post-weaning growth was observed for the colostrum fatty acid composition. Hence, the most significant associations were observed between ADWG increase and a decrease in saturated fatty acids (mainly C18:0), increase in the ratio C18:1/C18:0 and the desaturase indices of colostrum. Other authors have observed the relationship between colostrum intake and piglet growth [39]. Colostrum is the first source of energy for the piglet [40] and therefore changes in its composition could alter the growth pattern during lactation. In fact, it has been described that multiple factors, including environmental changes, could modify its metabolomic composition and therefore have effects on growth [41]. Previous investigations carried out in the scope of this same research project showed that the administration of VE or HXT modified the composition of colostrum and milk [22] in different ways. Thus, HXT increased the polyunsaturated fatty acid proportion of colostrum, whereas VE improved the n-7 monounsaturated [22]. Oleic acid is preferentially oxidated over linoleic acid of milk, and therefore monounsaturated fatty acids could serve as a faster energy source for growth and development [42]. In addition, oleic acid is one of the principal fatty acids of the brain phosphoglycerides, which could contribute to brain development [43]. Other fatty acids with a high rate of oxidation are n-3 [17,42], which could also affect head development post-weaning as observed in the present research by the linear and significant relationship between occipito-nasal length and n-3 proportion of colostrum. However, the increase in elongase C18 to C20 of colostrum, was negatively correlated with the weight of liver and gut in the present study. This could be explained because saturated medium chain fatty acids are oxidized easier and faster, whereas those of longer chains take longer to be available to be oxidized and could be stored in tissues for later use [42,44]. This agrees with the positive linear relationship observed between 20 days milk elongase C20–C18 and piglets’ body weight, trunk and head weight postweaning. On the other hand, the positive relationship observed between milk C18:3 n-3 and gut weight and the abdominal perimeter could be associated with an improved development at this level, since these long-fatty acids are incorporated into cell membrane phospholipids including the intestine, potentially improving gut health [44], but more research is needed to clarify this.

To our knowledge, this is the first study in which the contribution of the specific colostrum and milk fatty acids on the piglet’s growth is quantified. Hojgaard et al. [45] quantified the impact of milk composition on the piglet’s growth at weaning and reported that milk protein concentration explained 87% of the total variation in piglet gain; however, these authors did not evaluate the specific fatty acid profile. According to our results and after the evaluation of the regression equations, C18:1/C18:0 and saturated fatty acids of colostrum would explain 49 and 47% of the weight gain. However, milk composition contributed to a lower extent and only 21% of the variation of the sow’s ability to desaturate and elongate milk fatty acids would explain the piglet’s weight post-weaning.

The results of the present research suggest that since the VE-supplemented sows had a higher desaturation capacity [22], this group would give rise to a higher weight and growth pattern in the piglets. In addition, these results agree with the direct relationship observed between the plasmatic levels of VE of the sow and the ADWG of the piglets in the lactation period; as well as the piglet’s plasma vitamin E levels and ADWG until weaning.

## 5. Conclusions

In conclusion, the administration of VE or HXT from late gestation are effective antioxidants to improve the oxidative status of the sows and the piglets. Both antioxidants produce similar weights and development at birth. However, during lactation, VE seems to be a better supplement. Positive and linear relationships between the sow’s oxidative status and piglet development demonstrates the maternal effect. The highest contribution to piglet development was observed by the sow’s desaturase capacity on colostrum. This study would confirm that VE supplementation to the sow diet could be more adequate than HXT for the development during the first weeks of a piglet’s life.

## Figures and Tables

**Table 1:** Oxidative status of sows given α-tocopherol (VE: 30 vs. 100 mg/kg) or hydroxytyrosol (HXT: 0 vs. 1.5 mg/kg) from day 85 of gestation.

	Control ^1^	VE ^2^	HXT ^3^	VE + HXT ^4^	VE-30	VE-100	HXT-0	HXT-1.5	RMSE ^5^	*p* VE ^6^	*p* HXT	*p* VE + HXT
***Day 110 of gestation***												
SOD ^7^, U/mL	0.39	0.39	0.58	0.44	0.48	0.42	0.39 ^a^	0.51 ^b^	0.153	0.247	0.044	0.252
GSHt ^8^, µM	1.72	1.54	2.79	3.16	2.25	2.35	1.63 ^a^	2.97 ^b^	1.419	0.864	0.019	0.608
GSSG ^9^, µM	0.70	0.73	0.97	0.77	0.83	0.75	0.72	0.87	0.247	0.367	0.125	0.231
Free GSH ^10^, µM	1.02	0.80	1.82	2.39	1.42	1.60	0.91 ^a^	2.11 ^b^	1.349	0.729	0.028	0.447
α-Tocopherol, µg/mL	1.40	2.25	1.54	2.28	1.47 ^B^	2.26 ^A^	1.82	1.91	0.666	0.004	0.746	0.824
***Day 20 of lactation***												
SOD, U/mL	0.43	0.62	0.58	0.54	0.51	0.58	0.53	0.56	0.320	0.486	0.977	0.496
GSHt, µM	2.55	2.93	2.05	3.18	2.30	3.05	2.74	2.61	1.596	0.256	0.651	0.399
GSSG, µM	0.96	1.17	1.00	1.01	0.98	1.09	1.07	1.01	0.481	0.562	0.581	0.776
Free GSH, µM	1.70	1.76	1.05	2.17	1.37	1.96	1.78	1.61	1.231	0.265	0.620	0.186
α-Tocopherol, µg/mL	1.48	3.01	1.81	2.79	1.65 ^B^	2.90 ^A^	2.25	2.30	0.668	<0.001	0.532	0.143
MDA ^11^, mM	0.13	0.09	0.08	0.09	0.11	0.09	0.11	0.09	0.063	0.384	0.220	0.245

^1^ Control = 30 mg of α-tocopheryl acetate/kg feed + 0 mg/kg hydroxytyrosol; ^2^ VE = 100 mg of α-tocopheryl acetate/kg feed + 0 mg/kg hydroxytyrosol; ^3^ HXT = 30 mg of α-tocopheryl acetate/kg feed + 1.5 mg/kg hydroxytyrosol; ^4^ VE + HXT = 100 mg of α-tocopheryl acetate/kg feed + 1.5 mg/kg hydroxytyrosol; ^5^ RMSE = Root mean square error (pooled SD); ^6^ *p* = differences were statistically different when *p* < 0.05; ^7^ SOD = superoxide dismutase; ^8^ GSHt = total glutathione; ^9^ GSSG = oxidized glutathione; ^10^ free GSH = reduced glutathione; ^11^ MDA = malondyaldehyde concentration; ^a,b,A,B^ Letter with different superscript were statistically significant.

**Table 2:** Performances of sows and piglets from sows given different levels of α-tocopheryl acetate (VE) or hydroxytyroxol (HXT) from day 85 of gestation.

	Control ^1^	VE ^2^	HXT ^3^	VE + HXT ^4^	VE-30	VE-100	HXT-0	HXT-1.5	RMSE ^5^	*p* VE ^6^	*p* HXT	*p* VE + HXT
***Sows’ weights***												
At d 85, kg	120.86	129.09	125.20	129.75	123.03	129.42	124.97	127.47	29.307	0.309	0.690	0.769
At farrowing, kg	113.20	120.42	113.54	123.46	113.37	121.94	116.81	118.50	26.497	0.267	0.826	0.860
At weaning, kg	106.40	112.50	102.73	110.85	104.57	111.67	109.45	106.79	22.850	0.281	0.685	0.878
***Piglets’ weights***												
At birth, kg	1.24	1.39	1.38	1.45	1.31 ^B^	1.42 ^A^	1.31 ^b^	1.42 ^a^	0.241	<0.001	<0.001	0.135
At day 7, kg	2.40	2.51	2.55	2.68	2.47 ^B^	2.60 ^A^	2.46 ^b^	2.62 ^a^	0.434	0.010	0.001	0.842
At day 20, kg	4.50	4.71	4.56	4.40	4.53	4.56	4.61	4.48	0.875	0.792	0.190	0.062
At day 28, kg	5.27	5.65	5.40	5.35	5.34	5.50	5.46	5.38	1.096	0.177	0.501	0.083
At day 58, kg	13.82	13.20	13.49	12.87	13.66	13.03	13.51	13.18	3.690	0.267	0.553	0.999
ADWG (1–7 d) ^7^	0.16	0.16	0.17	0.18	0.16	0.17	0.16 ^b^	0.17 ^a^	0.042	0.231	0.042	0.195
ADWG (8–20 d)	0.14 ^b^	0.16 ^a^	0.14 ^b^	0.14 ^b^	0.14	0.15	0.15 ^a^	0.14 ^b^	0.039	0.137	0.001	0.017
ADWG (21–28 d)	0.11	0.13	0.10	0.12	0.10 ^B^	0.13 ^A^	0.11	0.11	0.057	<0.001	0.819	0.936
ADWG (0–28 d)	0.14	0.15	0.14	0.14	0.14 ^B^	0.15 ^A^	0.14	0.14	0.032	0.013	0.505	0.233
ADWG (29–58 d)	0.25	0.24	0.24	0.23	0.25	0.24	0.25	0.24	0.096	0.387	0.528	0.973

^1^ Control = 30 mg of α-tocopheryl acetate/kg feed + 0 mg/kg hydroxytyrosol; ^2^ VE = 100 mg of α-tocopheryl acetate/kg feed + 0 mg/kg hydroxytyrosol; ^3^ HXT = 30 mg of α-tocopheryl acetate/kg feed + 1.5 mg/kg hydroxytyrosol; ^4^ VE + HXT = 100 mg of α-tocopheryl acetate/kg feed + 1.5 mg/kg hydroxytyrosol; ^5^ RMSE = Root mean square error (pooled SD) ^6^ *p* = differences were statistically different when *p* < 0.05; ^7^ ADWG = Averaged daily weight gain; ^a,b,A,B^ Letters with different superscripts were statistically significant.

**Table 3:** Body measurements (cm) and weight (kg) of piglets from sows given α-tocopheryl acetate (VE: 30 vs. 100 mg/kg) or hydroxytyrosol (HXT: 0 vs. 1.5 mg/kg) from day 85 of gestation.

	Control ^1^	VE ^2^	HXT ^3^	VE + HXT ^4^	VE-30	VE-100	HXT-0	HXT-1.5	RMSE ^5^	*p* VE ^6^	*p* HXT	*p* VE + HXT
***At birth***												
Biparietal diameter	4.75	4.90	4.83	5.13	4.79 ^B^	5.01 ^A^	4.82 ^b^	4.98 ^a^	0.369	<0.001	<0.001	0.060
Body/Head ratio	0.26 ^b^	0.28 ^a^	0.29 ^a^	0.28 ^a^	0.27	0.28	0.27	0.28	0.042	0.042	0.007	0.003
Occipito-nasal length	13.10	13.83	12.99	13.83	13.05 ^B^	13.83 ^A^	13.46	13.41	0.927	<0.001	0.609	0.590
Trunk length	21.11 ^c^	22.06 ^b^	22.66 ^a^	22.63 ^a^	21.89	22.35	21.59	22.64	1.831	0.023	<0.001	0.014
Thoracic perimeter	23.22	23.86	24.05	24.51	23.63 ^B^	24.19 ^A^	23.54 ^b^	24.28 ^a^	1.748	0.004	<0.001	0.631
Abdominal perimeter	19.52	20.61	20.39	20.87	19.96 ^B^	20.74 ^A^	20.06 ^b^	20.63 ^a^	1.885	<0.001	0.006	0.147
***Post-weaning***												
Body weight	5.67	6.49	6.13	6.11	5.90	6.30	5.90	6.12	1.289	0.134	0.776	0.169
Body/Head ratio	7.73	8.08	7.74	8.19	7.74	8.13	7.74	7.97	0.180	0.083	0.958	0.188
Carcass weight	3.18	3.78	3.51	3.54	3.34	3.66	3.47	3.52	0.769	0.073	0.740	0.164
Bowels weight	1.65	1.88	1.67	1.67	1.66	1.78	1.75	1.67	0.581	0.348	0.719	0.300
Head weight	0.73	0.80	0.79	0.75	0.76	0.78	0.76	0.77	0.127	0.432	0.702	0.096
Loin weight	0.01	0.01	0.01	0.01	0.01	0.01	0.01	0.01	0.005	0.715	0.976	0.448
Liver weight	0.18	0.21	0.20	0.21	0.19	0.21	0.21	0.20	0.052	0.215	0.482	0.205
Kidneys weight	0.04	0.04	0.04	0.04	0.04	0.04	0.04	0.04	0.009	0.374	0.324	0.278
Gut weight	0.90	1.03	0.91	0.88	0.90	0.96	0.95	0.89	0.357	0.477	0.653	0.236
Biparietal diameter	5.97	6.08	6.25	6.09	6.11	6.08	6.12	6.17	0.294	1.000	0.084	0.176
Occipito-nasal length	15.94	15.95	16.30	16.24	16.12	16.09	16.22	16.27	0.986	0.827	0.171	0.827
Trunk length	42.25	43.75	41.65	42.24	41.95	42.99	42.02	41.94	4.497	0.291	0.478	0.551
Thoracic perimeter	37.94	39.55	39.32	39.07	38.63	39.31	39.27	39.20	3.487	0.199	0.727	0.229
Abdominal perimeter	29.91	31.85	31.34	31.80	30.62	31.83	31.90	31.57	3.278	0.168	0.356	0.221

^1^ Control = 30 mg of α-tocopheryl acetate/kg feed + 0 mg/kg hydroxytyrosol; ^2^ VE = 100 mg of α-tocopheryl acetate/kg feed + 0 mg/kg hydroxytyrosol; ^3^ HXT = 30 mg of α-tocopheryl acetate/kg feed + 1.5 mg/kg hydroxytyrosol; ^4^ VE + HXT = 100 mg of α-tocopheryl acetate/kg feed + 1.5 mg/kg hydroxytyrosol; ^5^ RMSE = Root mean square error (pooled SD); ^6^ *p* = differences were statistically different when *p* < 0.05; ^a,b,c,A,B^ Letters with different superscripts were statistically significant.

**Table 4:** Pearson correlation coefficients oxidative parameters of sows and piglets’ measurements post-weaning from sows given α-tocopheryl acetate (VE: 30 vs. 100 mg/kg) or hydroxytyrosol (HXT: 0 vs. 1.5 mg/kg) from day 85 of gestation.

Piglets Measurements Post-Weaning	Sow’s GSSG ^2^	Sow’s Plasma VE	Sow’s Plasma MDA ^3^
ADWG ^1^	−0.32	0.36 ^b^	−0.40 ^b^
Body weight	−0.41 ^b^	0.17	−0.37
Carcass weight	−0.35	0.31	−0.32
Bowels weight	−0.30	−0.06	−0.29
Loin weight	−0.34	−0.23	−0.40 ^b^
Head weight	−0.39 ^b^	0.00	−0.47 ^a^
Liver weight	−0.39 ^b^	0.08	−0.38 ^b^
Kidneys weight	−0.35	0.02	−0.40 ^b^
Gut weight	−0.32	−0.13	−0.25
Biparietal diameter	−0.48 ^a^	0.13	−0.45 ^b^
Occipito-nasal length	−0.46 ^b^	−0.13	−0.38 ^b^
Trunk length	−0.33	0.19	−0.26
Thoracic perimeter	−0.41 ^b^	0.11	−0.35
Abdominal perimeter	−0.41 ^b^	0.08	−0.35

^1^ ADWG = Averaged daily weight gain; ^2^ GSSG = oxidized glutathione; ^3^ MDA = malondyaldehyde concentration; ^a, b^ Letters with different superscripts were statistically significant; ^a^ red color *p* < 0.001; ^b^ blue color *p* < 0.05.

**Table 5:** Pearson correlation coefficients (r) and regression equations between colostrum composition and growth of post-weaning piglets born from sows given α-tocopheryl acetate (VE: 30 vs. 100 mg/kg) or hydroxytyrosol (HXT: 0 vs. 1.5 mg/kg) from day 85 of gestation.

	Intercept		s.d. ^12^	Slope		s.d.	Variable x	r	R^2^	*p* Linear ^13^
							***Colostrum Fatty Acid***			
ADWG ^11^, kg/d (0–28 d)	0.05	±	0.03	0.02	±	0.01	C16:1n-7	0.57	0.33	0.002
	0.28	±	0.04	−0.02	±	0.01	C18:0	−0.62	0.38	0.001
	0.05	±	0.03	0.03	±	0.01	C18:1n-7	0.48	0.23	0.012
	0.50	±	0.08	−0.01	±	0.00	∑SAT ^1^	−0.69	0.47	0.000
	0.04	±	0.02	0.69	±	0.17	C16:1n-7/C16:0	0.63	0.40	0.001
	−0.88	±	0.21	1.17	±	0.25	C18:1/C18:0	0.70	0.49	<0.001
	−0.27	±	0.11	0.68	±	0.18	Δ-9-desaturase ^2^	0.61	0.37	0.001
	0.07	±	0.03	2.60	±	1.11	Δ-6-desaturase ^3^	0.43	0.19	0.028
	0.34	±	0.07	−0.93	±	0.30	Elongase 18–16 ^4^	−0.52	0.27	0.005
Loin weight, g	−24.42	±	14.91	41.05	±	18.32	C20:1/C20:0	0.40	0.16	0.034
Liver weight, g	24.24	±	83.93	9.44	±	4.52	Elongase 16–14 ^5^	0.38	0.14	0.047
	251.93	±	20.68	−4156.73	±	1423.14	Elongase 20–18 ^6^	−0.50	0.25	0.007
	−249.25	±	158.21	551.14	±	194.49	C20:1/C20:0	0.49	0.24	0.009
Gut weight, g	1160.90	±	137.91	−19,594.60	±	9492.35	Elongase 20–18	−0.38	0.14	0.049
	−1346.78	±	1039.76	2776.78	±	1278.13	C20:1/C20:0	0.39	0.15	0.039
Occipital nasal length, cm	12.11	±	1.74	0.20	±	0.09	∑PUFA	0.41	0.17	0.032
	12.32	±	1.73	0.21	±	0.10	∑n-6	0.39	0.15	0.040
	12.71	±	1.13	2.87	±	0.96	∑n-3 ^7^	0.51	0.26	0.006
Trunk length, cm	16.35	±	11.78	0.51	±	0.24	∑MUFA ^8^	0.39	0.15	0.041
	16.35	±	11.78	0.51	±	0.24	∑PUFA ^9^	0.39	0.15	0.041
	58.29	±	7.53	−0.93	±	0.42	∑n-6 ^10^	0.40	0.16	0.035
Thoracic perimeter, cm	15.75	±	9.97	27.91	±	12.26	C20:1/C20:0	0.41	0.17	0.031
Abdominal perimeter, cm	20.82	±	4.39	0.56	±	0.24	Elongase 16–14	0.43	0.18	0.026
	9.37	±	9.51	26.44	±	11.69	C20:1/C20:0	0.41	0.16	0.032

^1^ ∑SAT = Sum of total saturated fatty acids; ^2^ Δ-9-desaturase index = (C14:1 + C16:1 + C18:1)/C14:0 + C14:1 + C16:0 + C16:1 + C18:0 + C18:1); ^3^ Δ-6-desaturase = (C18:3n-6 + C18:4n-3)/(C18:2n-6 + C18:3n-3 + C18:3n-6 + C18:4n-3); ^4^ Elongase (18/16) index = C18:0/C16:0; ^5^ Elongase (16/14) index = C16:0/C14:0; ^6^ Elongase (20/18) index = C20:0/C18:0; ^7^ ∑n-3 = Sum of total n-3 fatty acids; ^8^ ∑MUFA = Sum of total monounsaturated fatty acids; ^9^ ∑PUFA = Sum of total polyunsaturated fatty acids; ^10^ ∑n-6 = Sum of total n-6 fatty acids; ^11^ ADWG = average daily weight gain; ^12^ s.d. = standard deviation; ^13^ *p* = differences were statistically different when *p* < 0.05.

**Table 6:** Pearson correlation coefficients (r) and regression equations between day 20 milk composition and growth of post-weaning piglets born from sows given α-tocopheryl acetate (VE: 30 vs. 100 mg/kg) or hydroxytyrosol (HXT: 0 vs. 1.5 mg/kg) from day 85 of gestation.

	Intercept		s.d. ^1^	Slope		s.d.	Variable x	r	R^2^	*p* Linear ^2^
							***Day 20 Milk***			
Piglet’s weight postweaning, kg	4.61	±	0.62	61.96	±	24.78	Elongase 20–18 ^3^	0.45	0.21	0.020
Piglet’s weight postweaning, kg	11.03	±	2.53	−6.49	±	3.29	C20:1/C20:0	0.37	0.14	0.060
Trunk length, cm	38.91	±	1.64	140.85	±	63.38	Elongase 20–18	0.41	0.17	0.036
Abdominal perimeter, cm	21.32	±	4.80	13.88	±	6.63	C18:3 n-3	0.39	0.15	0.047
Head weight, kg	0.63	±	0.06	5.94	±	2.14	Elongase 20–18	0.49	0.24	0.011
Gut weight, kg	−0.39	±	0.50	1.84	±	0.69	C18:3 n-3	0.49	0.24	0.014

^1^ s.d. = Standard deviation of mean; ^2^ *p* = differences were statistically different when *p* < 0.05; ^3^ Elongase (20–18) index = C20:0/C18:0.

## Data Availability

Data are contained within the article.

## Appendix A

**Table A1:** Analyzed composition and fatty acid proportion of the experimental diets.

*Analysed Composition* ^1^	CONTROL	VE^2^	HXT	HXT + VE
Dry matter, %	90.8	89.8	91.1	91.2
Crude Protein, %	13.1	14.0	15.0	14.5
Fat, %	4.3	3.6	3.8	3.7
Ash, %	6.5	6.4	6.9	6.0
Fiber, %	4.3	4.4	4.3	4.4
Starch, %	49.0	46.1	41.1	43.0
Vitamin E, mg/kg	70.5	103.6	65.4	114.1
***Fatty acid composition***				
C14:0	0.60	0.66	0.56	0.60
C16:0	19.80	21.48	19.10	20.11
C16:1n-9	0.12	0.15	0.12	0.12
C16:1n-7	0.79	0.87	0.73	0.78
C18:0	4.77	4.80	4.45	4.54
C18:1n-9	28.97	25.26	30.33	26.94
C18:1n-7	1.92	1.49	1.57	1.60
C18:2n-6	38.97	40.81	38.97	40.89
C18:3n-3	3.04	3.44	3.15	3.36
C20:0	0.30	0.26	0.30	0.31
C20:1n-9	0.52	0.58	0.55	0.58
∑SAT	25.47	27.20	24.41	25.55
∑MUFA	32.33	28.35	33.29	30.02
∑PUFA	42.01	44.25	42.11	44.25

^1^ Ingredients (%): Corn: 16.03; wheat: 10; Barley: 50; soya meal 47: 6.73; wheat bran: 6.54; pork lard: 1; beetroot pulp: 5; calcium carbonate: 1.98; bicalcium phosphate: 0.54; salt: 0.5; L-lysine: 0.50; Methionine: 0.04; threonine: 0.18; choline chloride: 0.03; premix: 0.3 (per kg/diet: vitamin A: 12,000 IU; Vitamin D_3_: 1400 IU; Vitamin B_1_: 1.1 mg; Vitamin B_2_: 6 mg; Vitamin B_12_: 0.08 mg; Vitamin B_6_: 12 mg; Nicotinic acid: 21 mg; Biotin: 0.12 mg; Pantothenic acid: 12 mg; Vitamin K_3_: 1.1 mg; Choline chloride: 225 mg; Fe (ferrous carbonate): 60 mg; Cu (pentahydrate sulphate): 14.2 mg; Zn (oxide): 100 mg; Mn (monohydrate sulphate): 30 mg; I (potasium iodure): 0.8 mg; Se (sodium selenite): 0.3 mg. Calculated metabolizable energy (ME) based on ingredient composition = 3030 Kcal ME/kg.
